# Bone marrow mesenchymal stem cells-derived exosomes suppress miRNA-5189-3p to increase fibroblast-like synoviocyte apoptosis via the BATF2/JAK2/STAT3 signaling pathway

**DOI:** 10.1080/21655979.2022.2045844

**Published:** 2022-03-04

**Authors:** Yiqun Zhang, Bizhi Tu, Qi Sha, Jun Qian

**Affiliations:** Department of Spine Surgery, First Affiliated Hospital of Anhui Medical University, Hefei, Anhui, China

**Keywords:** Ankylosing spondylitis, bone marrow mesenchymal stem cells, exosome, fibroblast-like synoviocytes, miR-5189-3p, BATF2, JAK2/STAT3/signaling

## Abstract

Ankylosing spondylitis (AS) is characterized by inflammation of the sacroiliac joint and the attachment point of the spine. Herein, we aimed to investigate the effect of bone marrow mesenchymal stem cells (BMSCs)-derived exosomes on apoptosis of fibroblast-like synoviocytes (FLSs) and explored its molecular mechanism. Exosomes were isolated from BMSCs and verified by transmission electron microscope and nanoparticle tracking analysis. FLSs were isolated and co-incubated with BMSC exosomes. Cell apoptosis was assessed using terminal deoxynucleotidyl transferase-mediated dUTP nick-end labeling analysis and flow cytometry. The results showed that BMSC exosomes increased apoptosis of FLSs. MiR-5189-3p was downregulated, while basic leucine zipper transcription factor ATF-like 2 (BATF2) was upregulated in FLSs by treatment of BMSC exosomes. As a direct target of miR-5189-3p, BATF2 inactivates the JAK2/STAT3 pathway. MiR-5189-3p suppressed apoptosis of FLSs and BATF2 exerted an opposite effect. In conclusion, BMSCs-derived exosomes suppress miR-5189-3p to facilitate the apoptosis of FLSs via the BATF2/JAK2/STAT3 signaling pathway, which facilitates the understanding of the therapeutic effect of BMSCs on AS and the underlying molecular mechanism.

## Graphical abstract

**Figure:**
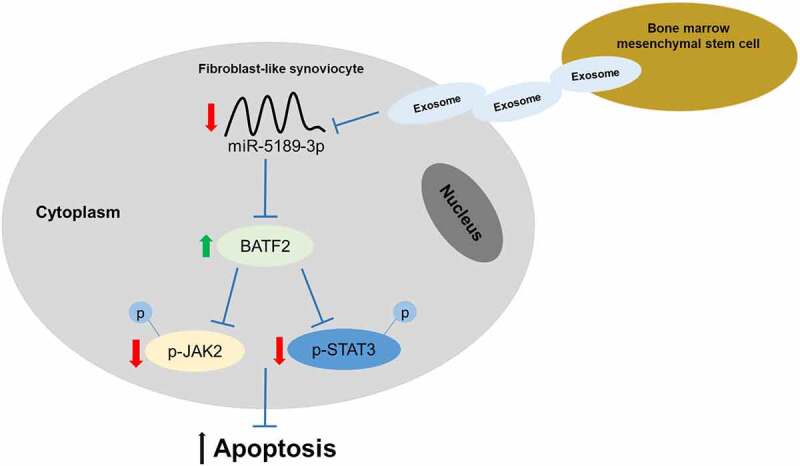

## Introduction

Ankylosing spondylitis (AS) is a systemic disease dominated by chronic inflammation of the axial joints [1]. The lesions mainly involve the spine and sacroiliac joints, and the disability will occur in the late stage. Since the initial stage of AS is relatively mild and insidious, most patients cannot be diagnosed early, leading to the delays in the condition and loss of the best opportunity for treatment [2]. The prevalence and the disability of the disease are relatively high. It has become a serious public health problem, which seriously affects the quality of life and labor ability of patients [3]. Therefore, early diagnosis and treatment of AS are the keys to the good prognosis of AS patients. However, the pathogenesis and regulatory mechanisms of the disease are currently unknown. Therefore, it is urgent to explore the molecular mechanism underlying AS, which will provide us a better understanding of the treatment of AS.

Exosomes, which could be secreted by various kinds of cells, are small vesicles ranging from 30 to 100 nm in size. Exosomes are now recognized to play important roles in cell-to-cell communication that contain proteins, lipids, as well as miRNAs [4,5]. Increasing numbers of studies have indicated that the mesenchymal stem cell (MSC)-derived exosomes involve in multiple physiology and pathology activities including osteogenesis, bone regeneration, osteoarthritis, inflammation, and tumorigenesis. For instance, exosomes derived from MSCs suppress chondrocyte degeneration in traumatic osteoarthritis [6]. Bone marrow mesenchymal stem cells (BMSCs)-derived exosomes, as drug carriers with specific functions, have great potential in hepatocellular carcinoma treatment in combination with anticancer drugs [7]. Moreover, BMSCs-derived exosomes attenuate hepatocyte apoptosis by activating autophagy *in vitro* [8]. Zuo *et al*. have reported that BMSC-derived exosomes alleviate radiation-induced bone loss [9]. These results indicate that BMSCs play crucial roles in the regulation and treatment of different diseases. Moreover, it is also showed that synovial exosomes play important roles in osteoclast differentiation in inflammatory arthritis, including rheumatoid arthritis, AS, gout, and osteoarthritis [10]. However, the effects of BMSC-derived exosomes on AS have not been well studied.

MicroRNAs (miRNAs) are small non-coding RNAs of 19–22 nucleotides and function by binding the sequence in the 3ʹuntranslated region (UTR) of the target messenger RNA (mRNAs) at post-transcriptionally level [11]. A large number of miRNAs have been proved to participate in the regulation of AS [12]. For example, miR-451 suppresses inflammatory responses in AS by targeting macrophage migration inhibitory factor [13]. MiR-214 stimulated by interleukin-17A regulates bone loss in AS patients [14]. Some miRNAs are also considered to be biomarkers of AS. Fotoh *et al*. have showed that miR-451a and miR-125a in AS have an important impact on disease diagnosis, prognosis, and outcomes [15].

It has been previously reported that miR-5189-3p was a newly identified miRNA in exosomes by RNA sequencing [16]. There is an increased risk of venous thromboembolism in the general ankylosing spondylitis population [17]. MiR-5189-3p showed significantly upregulated expression in blood samples from patients with deep vein thrombosis [18]. Thus, the present study focused on miR-5189-3p. We aimed to explore whether BMSC exosomes have effects on the apoptosis of FLSs and whether miR-5189-3p is regulated by BMSC exosomes and participates in AS. Several studies found that JAK2/STAT3 signaling play important roles in AS [19,20]. We hypothesized that this pathway is involved in the downstream pathway of BMSC exosomes-mediated miR-5189-3p in this study.

## Materials and methods

### Fibroblast-like synoviocyte (FLS) isolation and culture

Synovial tissue specimens of AS patients were collected from the orthopedics of the First Affiliated Hospital of Anhui Medical University. The studies were conducted in accordance with government policy and the Declaration of Helsinki, and the project was approved by the Ethics Committee of The First Affiliated Hospital of Anhui Medical University (PJ2021-16-12). A copy of the ethical approvement was provided in supplementary materials S1. The FLS was isolated from the synovial tissues as previously reported [21]. Briefly, the synovial tissues were cut into about 1–3 pieces and detached with 1% collagenase I for 2 h, after washing with Dhank’s balanced salt solution 2–3 times. The tissue pieces were continuously cultured in a pre-warmed complete Dulbecco’s Modified Eagle Medium (DMEM, 12,430,054, Gibco, USA) containing 2 mM of glutamine, 25 mM of 4-(2-hydroxyethyl)-1-piperazine’ethanesulfonic acid and 10% fetal bovine serum (FBS, Gibco, USA). Next, they were detached with 0.25% ethylene diamine tetraacetic acid-trypsin when the primary cells were basically fused into pieces. Cells were passaged at 1:3 and those at passage 3 were identified by flow cytometry, hematoxylin-eosin (H&E) staining and immunohistochemistry (IHC), respectively. For H&E staining, FLS were fixed with ethanol, processed by H&E staining and dehydrated with gradient ethanol and the cell morphology was observed under a microscope. For IHC, the cells were fixed in methanol at −20°C and sealed with 0.3% H_2_O_2_-formaldehyde. Next, the coverslips were supplemented with mouse anti-human Vimentin (1:250, Abcam ab92547), and cells incubated with PBS acted as a negative control. Then cells were incubated with secondary antibody (ZSGB-BIO, PV-9001) and detected by a biotin-avidin reaction system (LSAB kit, DAB reaction system ZSGB-BIO, ZLI-9018) according to the manufacturer’s instructions and the cells were observed under a microscope.

### Isolation, culture and identification of human BMSCs

A total of 4 mL of bone marrow fluids were obtained from AS patients in the orthopedics of the First Affiliated Hospital of Anhui Medical University. Samples were mixed with 4 mL of DMEM supplemented with 10% FBS and centrifuged at 1000 rpm for 20 min. Cells were resuspended by lymphocyte separation solution and were centrifuged at 2000 rpm/min for 20 min and the supernatant was discarded. Next, the cells were resuspended and cells with density of 1 × 10^6^ cells/mL were cultured in the 90% DMEM supplemented with 10% FBS, 100 μg/mL penicillin and 100 μg/mL streptomycin at 37°C in a 5% CO_2_-contained incubator under 95% saturation humidity. Subculture was performed when the cell confluence reached 80%. Cell purity identification was detected by cell morphology observation under the microscope. CD44 expression was analyzed by flow cytometry analysis. Flow cytometry raw data (.fcs files) and the gating strategy were provided in supplementary materials S2.

### Extraction and identification of exosomes from BMSCs

Exosomes were extracted from BMSCs and purified by Total Exosome Isolation Reagent (4,478,359, Invitrogen, USA). Morphology of exosomes was identified by transmission electron microscopy (TEM). Briefly, exosomes were diluted to 500 μg/ml and fixed with 2.5% glutaraldehyde for 2 h. Approximate 10 μL of exosome suspension was placed on formvar carbon-coated 300-mesh copper electron microscopy grids (Agar Scientific Ltd., Stansted, UK), and incubated for 5 min at room temperature. The grids were washed three times with PBS, air dried for 5 min, and imaged under TEM (HT7700; HITACHI, Tokyo, Japan) at 80 kV. Nanoparticle tracking analysis (NTA) was detected by ZetaView PMX 110 (Particle Metrix, Germany). The diameter and concentration were calculated by Stocker-Einstein equation and the data was analyzed by Zetasizer software (Malvern Instruments). FLSs were cultured to 70% confluence on a sterile 6-well culture plate at 5 × 10^5^–1 × 10^6^ cells/well. The BMSCs exosomes were co-incubated with FLSs for 24 h.

### Cell transfection

MiR-5189-3p mimics and inhibitors were used for overexpressing and silencing endogenous miR-5189-3p expression, respectively, and were transfected into FLSs at a final concentration of 50 nM by Lipofectamine 2000 reagent (Invitrogen, Carlsbad, CA, USA. For basic leucine zipper transcription factor ATF-like 2 (BATF2) function analysis, the BATF2-pcDNA3.1 and sh-BATF2-pcDNA3.1 were transfected into FLSs at a final concentration of 100 nM by Lipofectamine 2000 reagent. After transfection for 48 h, cells were harvested for further experiments. Sequences of BATF2 and sh-BATF2 that were inserted into the pcDNA3.1 vector were shown in Supplementary materials S3.

### Apoptosis analysis

Analysis of apoptotic cells was performed using the terminal deoxynucleotidyl transferase-mediated dUTP nick-end labeling (TUNEL) staining kit following the manufacturer’s instruction (Keygen biotech, KGA7072). Briefly, FLSs from different groups were grown on glass coverslips and transfected cells were then washed with PBS, fixed with 4% paraformaldehyde in PBS, and incubated with primary antibodies. TUNEL-positive cells had a pyknotic nucleus with dark green fluorescence staining. The nuclei were counterstained with Hoechst 33,258 (Beyotime, C1017). Images of the sections were taken using a fluorescence microscope (OLYMPUS BX51, Japan). Flow cytometry analysis of cell apoptosis was also performed according to the manufacturer’s guidelines. Briefly, cells were incubated with prodium iodide (PI) and Annexin V-fluorescein isothiocyanate (FITC) in the darkness at room temperature. Flow cytometric analysis was immediately performed. Flow cytometry raw data (.fcs files) and the gating strategy were provided in supplementary materials S2.

### Quantitative reverse transcription-polymerase chain reaction (qRT-PCR)

TRIzol reagent (Biosharp, BS259A) was used to extract the total RNA from FLSs. A total of 2 μg of RNA was used to synthesize cDNA using the RevertAid First Strand cDNA Synthesis Kit (Vazyme, R223-01) and miDETECT A TrackTM miRNA qRT-PCR starter Kit (Vazyme, MR101-01/02) according to the manufacturer’s instructions. qRT-PCR was performed using an ABI 7500 instrument (Roche LightCycler® 480II, USA) in a 20 μL of reaction volume including 10 μL of SybrGreen qPCR master Mix, 0.5 μL of each primer (10 μM), 1 μL of the cDNA template, and 8 μL of ddH_2_O. Amplification processes were as follows: 95°C, 2 min; 94°C, 20s and 60°C, 20s and 72°C, 20s for 40 cycles. The relative expression of miRNAs was normalized to U6, and mRNAs were normalized to actin. Relative miRNA and mRNA expression levels were calculated by the 2^−ΔΔCt^ method [22]. Primers were synthesized by Tsingke Biotechnology Co., Ltd (Beijing, China) and the detailed information was listed in Supplementary materials S4.

### Western blotting

Total protein was extracted from FLSs and BMSC exosomes using RIPA buffer (Biosharp, BL521A) containing protease inhibitors (Thermo, A32955) following the manufacturer’s instructions. Proteins were quantified using the BCA™ Protein Assay Kit (Biosharp, BL521A). A total of 30 μg of proteins were separated in 10–12% sodium dodecyl sulfate polyacrylamide gel electrophoresis and transferred onto polyvinylidene fluoride membranes. The primary antibody solution was prepared in 5% blocking buffer. Primary antibodies including anti-BATF2 (16,592-1-AP, 1:1000, Proteintech), anti-STAT3 (ab68153, 1:1000, Abcam), anti-Alix (ab134045, 1:1000, Abcam), anti-CD9 (ab236630, 1:1000, Abcam), and anti-CD63 (ab134045, 1:2000, Abcam), anti-p-STAT3 (phospho Y705, ab267373, 1:1000, Abcam), anti-JAK2 (ab108596, 1:1000, Abcam), anti-p-JAK2 (phospho Y1007 + Y1008, ab32101, 1:2000, Abcam) and anti-GAPDH (ab8245, 1:5000, Abcam) were incubated with the membrane at 4°C overnight, followed by a brief wash and incubation with secondary antibody anti-mouse IgG (BA1051, 1:10,000, BOSTR) or anti-rabbit IgG (BA1054, 1:15,000, BOSTR) for 1 h at room temperature. The immuno-complexes were finally detected by ECL after washing by TBST and the bands were analyzed using the ImageJ software. The uncorrupted full size Western blot images were provided in supplementary materials S5.

### Animal experiments

SKG mice were initially used as a model of rheumatoid arthritis, while, after system injection of curdlan, SKG mice developed the clinical characteristics of AS [23]. Female SKG mice aged 10 weeks were purchased from Clea Japan (Tokyo, Japan) and housed in individually ventilated cages and provided with water and standard diet *ad libitum* under a specific pathogen-free facility. All animal experiments were approved by the Institutional Animal Care and Use Committee of the First Affiliated Hospital of Anhui Medical University and conducted under the Laboratory Animals Welfare Act, Guide for the Care and Use of Laboratory Animals. There were six groups: negative control (*n* = 10 mice), AS group (*n* = 10 mice), AS + exosome group (*n* = 10 mice), miR-5189-3p knockout (KO) group (*n* = 10 mice), AS + miR-5189-3p-KO group (*n* = 10 mice), and AS + exosome + miR-5189-3p-KO group (*n* = 10 mice). For induction of a mouse model of AS, a suspension of curdlan was intraperitoneally injected into SKG mice at the dose of 3 mg/kg on day 3. Mice in the AS + exosome group or AS + exosome + miR-5189-3p-KO were intraperitoneally injected with 100 μL of PBS containing 50 μg of exosomes twice a week beginning from day 7 [24]. For silencing of miR-5189-3p, mice received an intra-articular injection of 5 μl of AAV-miR-5189-3p (5 × 10^9^ viral particles/μL; Genechem Biotech Inc, Shanghai, China) on day 0. Clinical signs of mice were observed by two independent researchers. Symptom scores of the affected joints were summed according to a previous study [25] with sixteen points as the highest possible points. At the endpoint (day 42) of animal experiments, ankle tissues were dissected from mice. Slices at a thickness of 3.5 µm were subjected to H&E staining. Pathologic scoring for enthesitis was conducted as previously described [26].

### Luciferase reporter assay

Binding site of miR-5189-3p on the 3ʹUTR of BATF2 was predicted from TargetScan (http://www.targetscan.org/vert_71/). Binding site of miR-5189-3p on MALAT1 was predicted from LncBase V2 [27]. The wild-type (WT) and mutant (MUT) 3ʹUTR of BATF2 were cloned into the pmirGLO luciferase vector to produce BATF2-WT and BATF2-MUT vectors. MiR-5189-3p sequences were also subcloned into the pmirGLO luciferase vector to produce pmirGLO-miR-5189-3p vector. For luciferase reporter assays, BATF2-WT or BATF2-MUT vectors and miR-5189-3p mimics were co-transfected into FLSs using Lipofectamine 2000 (Invitrogen, Thermo Fisher Scientific). The pmirGLO-miR-5189-3p vector was co-transfected with metastasis associated lung adenocarcinoma transcript 1 (MALAT1) expressing vector into FLSs using Lipofectamine 2000. Luciferase activity was detected using the Dual Luciferase Reporter Assay System (Promega, Madison, WI, USA) after 48 h of transfection.

### Statistical analysis

All statistical analysis was performed using the SPSS 18.0 software, and the graphs were generated using GraphPad Prism 6.0 (GraphPad Software, San Diego, CA, USA). Data were shown as the mean ± standard error of mean (SEM) from at least three independent experiments. Statistical comparisons were performed using unpaired t test and one-way analysis of variance (ANOVA) between two groups and more than two groups, respectively. Variables were analyzed at different time points using Bonferroni-corrected repeated measures ANOVA. *P* < 0.05 was considered as a level of statistical significance.

## Results

### Exosomes isolation from BMSCs

**Figure 1.**
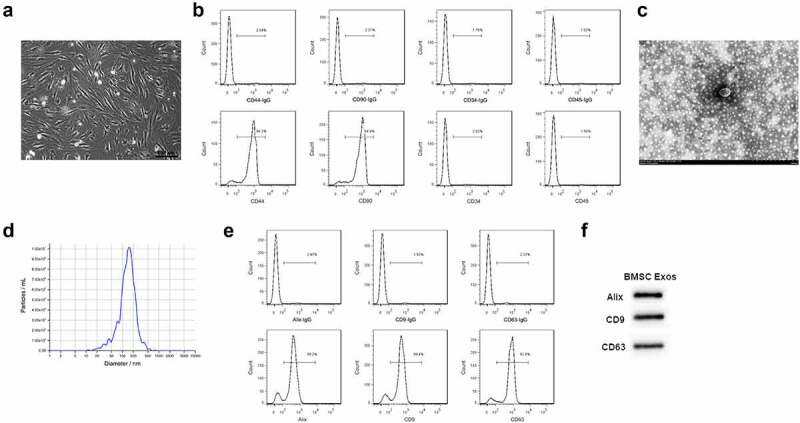
**Exosomes isolation from BMSCs**. (a) The morphology of BMSCs after subculture. Bar = 200 μm. (b) CD44, CD90 positive cells and CD34 and CD45 negative cells detected by flow cytometry. (c) Exosomes isolated from BMSCs detected by TEM. Bar = 150 nm. (d) Particle diameter of exosomes isolated from BMSCs detected by NTA analysis. (e) Flow cytometry analysis for Alix, CD9 and CD63 in the isolated exosomes of BMSCs. (f) Western blotting results for Alix, CD9 and CD63 in the isolated exosomes of BMSCs.

To know the effect of BMSCs exosomes on FLSs, BMSCs were isolated, purified, and validated. As shown in Figure 1a, all the human BMSCs adhered to the wall and showed uniform spindle cell-like growth after subculture for 24 h. The rates of CD90 and CD44 positive cells were 94.9% and 94.2% while CD34 and CD45 negative cells were 2.52% and 1.5%, respectively (Figure 1b), suggesting that human BMSCs were successfully isolated and purified. Moreover, the exosomes presented a typical exosomal double-layer capsule ultrastructure with saucer-shaped or hemispherical concave on one side (Figure 1c). We found that the peak particle diameter of the BMSCs exosomes ranged from 100 nm to 200 nm (Figure 1d). In addition, the expression of Alix, CD9 and CD63 showed high expression in the isolated human BMSCs exosomes, as revealed by the flow cytometry and Western blotting results (Figure 1e-f).

### BMSCs exosomes promote apoptosis of FLSs

**Figure 2.**
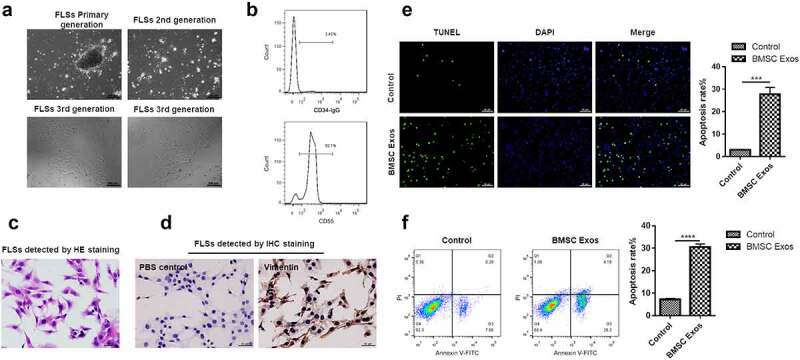
**BMSCs exosomes promoted the apoptosis of FLSs**. (a) Optical microscope phenotype of primary FLSs routinely cultured to the third passage identified by the inverted microscope. Bar = 200 μm. (b) Flow cytometry was used to detect the CD55 positive cells. (c) H&E staining analysis for synoviocyte nucleus located in the center of cells, with reddish cytoplasm. (d) Immunohistochemical staining to detect the FLSs, the positive cells were stained brown, which was indicated by Vimentin, and the control group using PBS showed no staining in the cytoplasm. (e) TUNEL to detect the percentage of FLSs occurring apoptosis under treatment with BMSCs exosomes. (f) Flow cytometry analysis was used to detect the apoptosis rate of FLSs under treatment with BMSCs exosomes. The data were expressed as mean ± SEM, ***p < 0.001, ****p < 0.0001, Exos group vs control group.

To know whether BMSCs exosomes functioned in the apoptosis of FLSs, exosomes from BMSCs were co-incubated with FLSs. The results suggested that primary FLSs were arranged in a long fusiform shape (Figure 2a) and more than 92% of the cells were FLSs detected by CD55 for 3 passages (Figure 2b). Furthermore, the synoviocyte nucleus was located in the center of cells, with reddish cytoplasm (Figure 2c), and almost all the cytoplasm was stained brown, which was indicated by Vimentin (Figure 2d). Therefore, FLSs at passage 3–5 were selected for subsequent experiments. We found that the number of FLSs showing apoptosis was dramatically increased under incubation with BMSCs exosomes compared with the control group (Figure 2e). Additionally, the apoptosis rate of FLSs was greatly increased under incubation with BMSCs exosomes (figure 2f), suggesting that BMSCs exosomes can promote apoptosis of FLSs.

### BMSCs exosome suppresses miR-5189-3p, which inhibits apoptosis of FLSs

**Figure 3.**
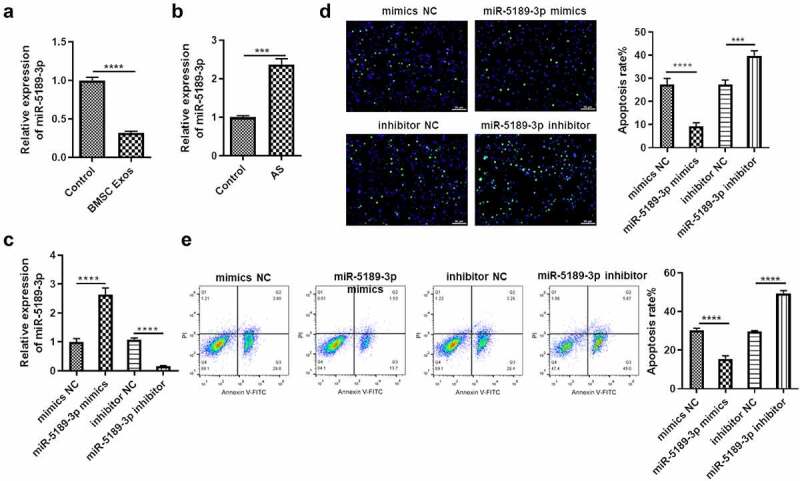
**BMSCs exosome suppresses miR-5189-3p, which inhibits apoptosis of FLSs**. (a) qRT-PCR was used to detect the expression of miR-5189-3p in the Exo group and control group. (b) qRT-PCR was used to detect the expression of miR-5189-3p in AS patients and control individuals. (c) qRT-PCR analysis showed the expression of miR-5189-3p in FLSs treated with miR-5189-3p mimics and inhibitor. U6 acted as control. (d) TUNEL analysis was used to detect the percentage of FLSs occurring apoptosis in FLSs after treatment with miR-5189-3p mimics and inhibitor. (e) Flow cytometry was used to detect the apoptosis of FLSs after treatment with miR-5189-3p mimics and inhibitor. The data were expressed as mean ± SEM, ***p < 0.001, ****p < 0.0001, miR-5189-3p mimics group or miR-5189-3p inhibitor group vs NC group.

Whether miR-5189-3p has effects on the apoptosis of FLSs and whether it is regulated by BMSC exosomes were explored. MiR-5189-3p showed significant downregulation in FLSs by treatment of BMSC exosomes (Figure 3a) and was upregulated in AS patients (Figure 3b), suggesting that miR-5189-3p might play an important role in FLSs. The function of miR-5189-3p was explored by transfecting FLSs with miR-5189-3p mimics or inhibitor. Expression of miR-5189-3p was successfully overexpressed or suppressed by miR-5189-3p mimics or inhibitor (Figure 3c). In addition, the number of FLSs occurring apoptosis was dramatically decreased in the miR-5189-3p mimics group while increased in the miR-5189-3p inhibitor group compared with the control group (Figure 3d-e). Moreover, miR-5189-3p mimics rescued the stimulative effects of BMSC exosomes on FLS apoptosis, while miR-5189-3p inhibitor further enhanced the BMSC exosomes-stimulated apoptosis of FLSs (Supplementary Figure 1A).

### BMSCs exosomes release MALAT1 to suppress miR-5189-3p expression

A previous study revealed that MALAT1 is released by BMSC exosomes [28]. We found that MALAT1 expression is higher in BMSC exosomes than BMSCs (Supplementary Figure 1B). Treatment of BMSC exosomes caused the upregulation of MALAT1 in FLSs (Supplementary Figure 1C). MALAT1 can decrease miR-5189-3p expression in FLSs (Supplementary Figure 1D). Results of the luciferase reporter assay revealed that the luciferase activity of pmirGLO-miR-5189-3p was suppressed by MALAT1 (Supplementary Figure 1E).

### BATF2 is a direct target of miR-5189-3p and is upregulated by BMSC exosomes

**Figure 4.**
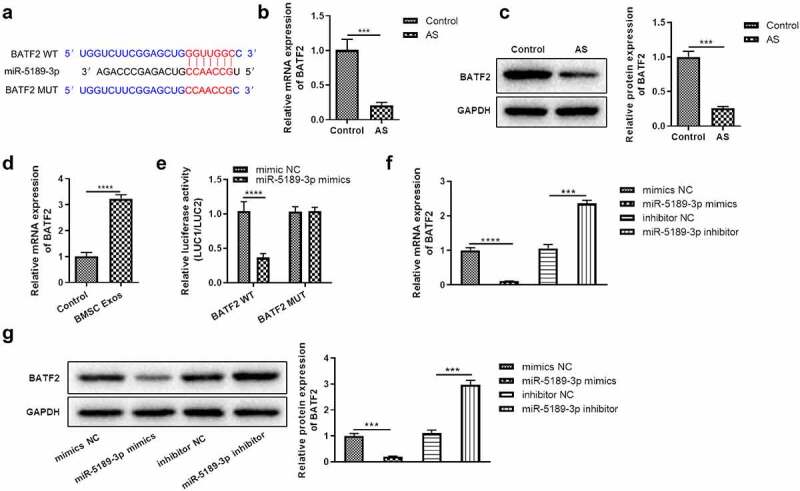
**BATF2 was the direct target of miR-5189-3p**. (a) The binding of miR-5189-3p to BATF2 3ʹUTR was predicted by TargetScan 7.2. (b-c) The expression of BATF2 in AS patients was detected by qRT-PCR and Western blot. ***p < 0.001, AS group vs control group. (d) The expression of BATF2 in FLSs after treatment with BMSC exosomes was detected by qRT-PCR. ****p < 0.0001, BMSC Exos group vs control group. (e) Dual luciferase reporter assays to detect the regulatory function of miR-5189-3p to BATF2, the relative luciferase activity was shown as firefly LUC activity/Renilla LUC activity. The data were expressed as mean ± SEM. ****p < 0.0001, pmiGLO-BATF2-WT or pmiGLO-BATF2-MUT group vs control group. (f-g) The expression of BATF2 in the miR-5189-3p mimics and inhibitor group by qRT-PCR and Western blot, respectively. ***p < 0.001, ****p < 0.0001, miR-5189-3p mimics group or miR-5189-3p inhibitor group vs NC group.

It has been revealed that miRNAs can exert effects by targeting 3ʹUTR of mRNAs. We investigated the targets of miR-5189-3p. BATF2 was predicted as a possible target gene of miR-5189-3p, and the binding site of miR-5189-3p on BATF2 3ʹUTR was predicted from TargetScan (Figure 4a). First, it was showed that the expression of BATF2 was significantly downregulated in AS tissues (Figure 4b,c). BMSC exosome enhanced BATF2 expression in FLSs (Figure 4d). Compared with the NC group, miR-5189-3p mimics significantly decreased the intensity of luciferase activity in the BATF2-WT group. However, miR-5189-3p mimics has no significant effect on the luciferase activity intensity in the BATF2-MUT group (Figure 4e). In addition, we found that the expression of BATF2 was significantly downregulated in the miR-5189-3p mimics group while upregulated in the miR-5189-3p inhibitor group compared with the NC group (figure 4f, g). These results suggested that BATF2 was the target gene of miR-5189-3p.

### BATF2 increases apoptosis of FLSs

**Figure 5.**
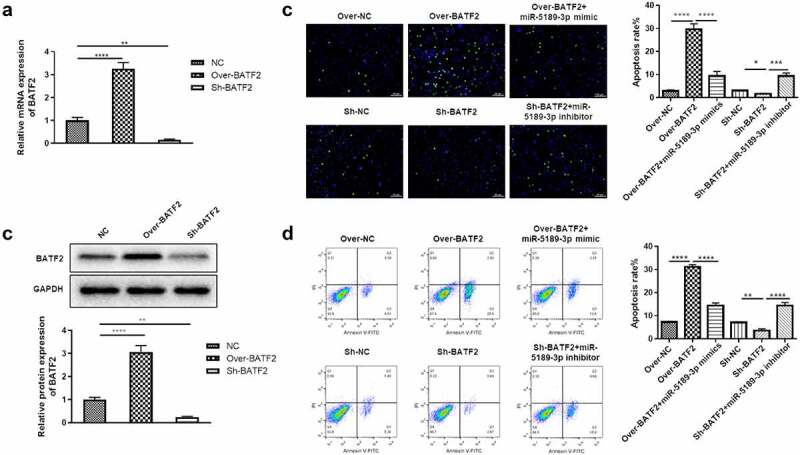
**BATF2 increases the apoptosis of FLSs**. (a) qRT-PCR analysis was used to detect the expression of BATF2 in the Over-BATF2 group and Sh-BATF2 group. (b) Western blot was used to detect the expression of BATF2 in the Over-BATF2 group and Sh-BATF2 group. GAPDH was set as the control. (c) TUNEL analysis to detect the percentage of apoptotic FLSs in the Over-NC, Over-BATF2, Over-BATF2+ miR-5189-3p mimic, Sh-NC, Sh-BATF2, and Sh-BATF2+ miR-5189-3p inhibitor groups. (d) Flow cytometry was used to detect the apoptosis rate of FLSs. The data were expressed as mean ± SEM. *p < 0.05, **p < 0.01, ***p < 0.001, ****p < 0.0001, Over-BATF2 group or Sh-BATF2 group vs NC group; Over-BATF2+ miR-5189-3p mimics group vs Over-BATF2 group, Sh-BATF2+ miR-5189-3p inhibitor group vs Sh-BATF2 group.

In order to know the effect of BATF2 on the apoptosis of FLSs, loss- and gain-of-function assays were performed after overexpressing or silencing BATF2 in FLSs. It was shown that the expression of BATF2 was significantly elevated in the Over-BATF2 group while dramatically decreased in the Sh-BATF2 group compared with the control group (Figure 5a). These results were also confirmed at the protein level (Figure 5b). Additionally, the number of FLSs occurring apoptosis was dramatically increased in the Over-BATF2 group while decreased in the Sh-BATF2 group compared with the control group (Figure 5c). Flow cytometry indicated that overexpressing BATF2 greatly promoted apoptosis of FLSs while the apoptosis of FLSs was significantly alleviated when suppressing BATF2 (Figure 5d). In addition, it was demonstrated that the BATF2-mediated apoptosis of FLSs was partially reversed by miR-5189-3p (Figure 5c,d).

### BATF2 affects the expression of p-JAK2 and p-STAT3

**Figure 6.**
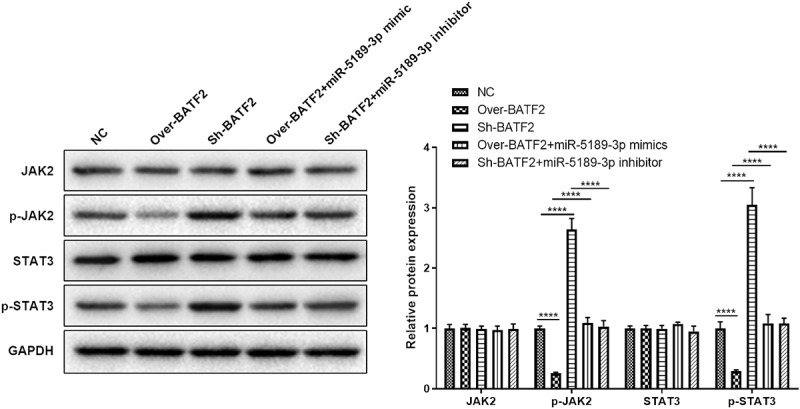
**BATF2 affected the expression of p-JAK2 and p-STAT3**. The expression of JAK2, STAT3 and the phosphorylated JAK2 and STAT3 was detected by Western blot in FLSs in the Over-NC, Over-BATF2, Over-BATF2+ miR-5189-3p mimic, Sh-NC, Sh-BATF2, and Sh-BATF2+ miR-5189-3p inhibitor groups. GAPDH was set as an internal control. Grey density was analyzed by the ImageJ tool. The data were expressed as mean ± SEM. ****p < 0.0001, Over-BATF2 group or Sh-BATF2 group vs NC group; Over-BATF2+ miR-5189-3p mimics group vs Over-BATF2 group, Sh-BATF2+ miR-5189-3p inhibitor group vs Sh-BATF2 group.

The JAK2/STAT3 signaling pathway has been demonstrated to play essential roles in various kinds of biological process, including cell proliferation, apoptosis and migration. In order to know whether BATF2 affects the JAK2/STAT3 signaling pathway, the protein expression of JAK2, STAT3 and the phosphorylated JAK2 and STAT3 was measured in BATF2 overexpressing or suppressing FLSs. The results showed that the expression of total JAK2 and STAT3 was not changed. However, the expression of p-JAK2 and p-STAT3 was significantly decreased in the Over-BATF2 group while upregulated in the Sh-BATF2 group compared with the control group. In addition, the BATF2-mediated expression of p-JAK2 and p-STAT3 was partially reversed by miR-5189-3p (Figure 6). These results demonstrated that BATF2 could activate the JAK2/STAT3 signaling pathway in FLSs, and its effect was controlled by miR-5189-3p.

### Effects of exosomes and miR-5189-3p in a mouse model of AS

**Figure 7.**
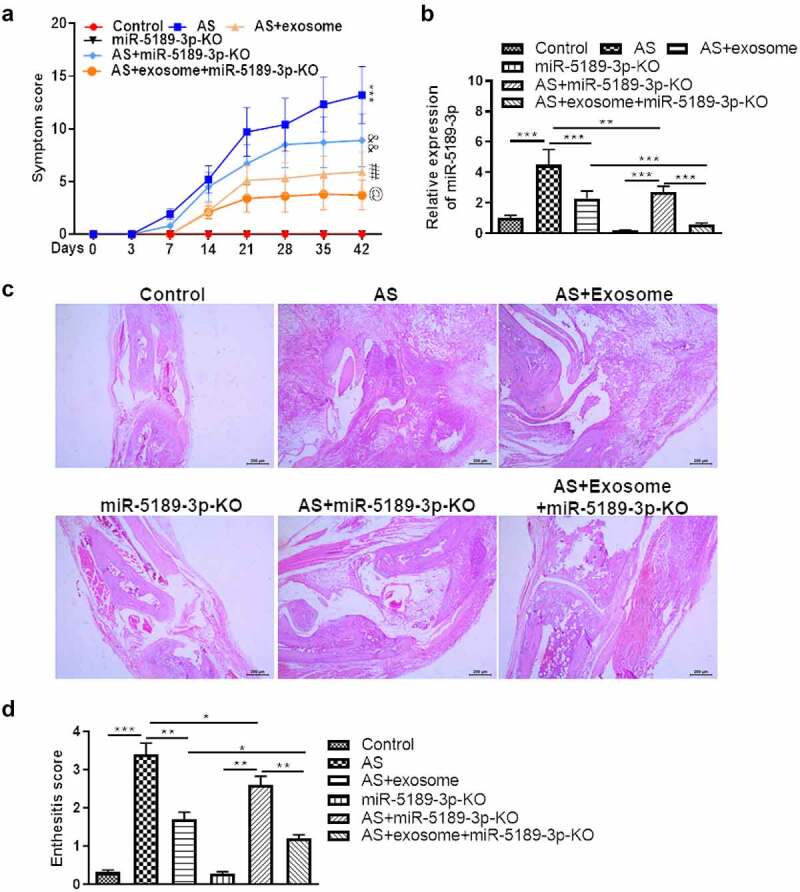
**Effects of exosomes and miR-5189-3p in a mouse model of AS**. (a) The arthritis scores were determined based on clinical arthritis severity of mice in each group. ***p < 0.001, AS group vs control group; ^##^*p* < 0.01, AS + exosome group vs AS group; ^&&^*p* < 0.01, AS+miR-5189-3p-KO group vs AS group; ^@^*p* < 0.05, AS + exosome + miR-5189-3p-KO vs AS + exosome group. (b) qRT-PCR analysis was used to detect the expression of miR-5189-3p in the ankle tissues of mice. (c) Representative H&E staining of ankle tissues are shown. (d) Analysis of histological scores for enthesitis was shown in bar graphs. *p < 0.05, **p < 0.01, ***p < 0.001.

Treatment of exosome or AAV-miR-5189-3p significantly reduced the severity of arthritis. Combination of exosome and AAV-miR-5189-3p treatment had a more significant effect on reducing arthritis severity than exosome or AAV-miR-5189-3p alone (Figure 7a). Expression of miR-5189-3p was higher in the ankle tissues of AS mice than control mice. Injection of AAV-miR-5189-3p successfully reduced miR-5189-3p expression. Injections of exosomes significantly decreased miR-5189-3p expression (Figure 7b). Histological analysis showed that mice treated with exosome or AAV-miR-5189-3p had less enthesitis than control mice. Combination of exosome and AAV-miR-5189-3p treatment had a more significant effect on reducing enthesitis than exosome or AAV-miR-5189-3p alone (Figure 7c-d).

## Discussion

In the present study, we found that BMSCs exosomes promoted the apoptosis of FLSs, suggesting that BMSCs exosomes play essential roles in the development of AS. The *in vivo* findings revealed that BMSCs exosomes reduced arthritis severity and enthesitis in a mouse model of AS. The exosomes derived from BMSCs have shown great potential in the treatment of diseases, such as enhancing the osteogenesis, ameliorating pain via abrogation of aberrant nerve invasion in subchondral bone in lumbar facet joint osteoarthritis and enhancing tendon-bone healing by regulating macrophage polarization [^29–31^]. Apoptosis induction of FLSs is an important molecular mechanism in treating AS [32], suggesting that BMSCs exosomes can inhibit the development of AS by promoting apoptosis of FLSs.

Increasing number of studies have demonstrated that MSCs exosome-mediated miRNAs play essential roles in regulating the status of FLSs. For example, exosomal miR-320a derived from MSCs regulates FLS activation by suppressing C-X-C motif chemokine ligand 9 expression [33]. MSC-originated exosomal long noncoding RNA heart and neural crest derivatives expressed 2 antisense RNA 1 impairs FLS activation by suppressing miR-143-3p in rheumatoid arthritis [34]. In the present study, miR-5189-3p was downregulated in the FLSs by treatment of BMSCs exosomes. Further investigations revealed that BMSCs exosomes release MALAT1 to bind with miR-5189-3p and to repress its expression. MiR-5189-3p overexpression suppresses the apoptosis of FLSs, which suggested that miR-5189-3p has the potential to contribute to the development of AS. Moreover, silencing of miR-5189-3p reduced arthritis severity and enthesitis in a mouse model of AS, indicating the harmful effect of miR-5189-3p in AS.

It has been demonstrated that miRNAs participate in many biological processes in the development of AS by targeting downstream genes [35,36]. In the present study, we found that BATF2 was a direct target of miR-5189-3p. Accumulating evidence has revealed that the transcription factor BATF2 has unique transcriptional activities, including regulating cytokines via TLR signals in macrophages, which affect mortality due to infection and cancer. Many studies have revealed that BATF2 can participate in the regulation of cell apoptosis [37,38]. In the present study, we found that the expression of BATF2 was significantly downregulated in AS patients. BMSC exosomes increased BATF2 expression in FLSs. Functional assays showed that overexpression of BATF2 promoted apoptosis of FLSs, and its effect was rescued by miR-5189-3p. These results demonstrated that overexpression of BATF2 can suppress the development of AS via promoting the apoptosis of FLSs.

Several studies revealed that the JAK2/STAT3 signaling plays an important role in AS [20,39,40]. Janus kinases 1/2/3 along with STAT signaling and signal transducers are responsible for the transmission of pro- and anti-inflammatory cytokine signals [41]. The JAK-mediated signaling transduction plays an essential role in bone development and metabolism [42]. Since AS is featured by altered osteoblastic differentiation and extensive inflammation, drugs targeting JAK, such as tofacitinib, have been used. In this context, targeting JAK in AS is now being tested in clinical trials [43]. In the present study, we found that the p-JAK2/STAT3 expression was significantly downregulated when overexpressing BATF2 while upregulated when suppressing BATF2. Moreover, the BATF2-mediated increased expression of p-JAK2 and p-STAT3 was partially reversed by miR-5189-3p in FLSs. These results demonstrated that BATF2 can inactivate the JAK2/STAT3 signaling pathway in FLSs, and its effect was regulated by miR-5189-3p.

## Conclusion

BMSCs exosomes promote the apoptosis of FLSs. MiR-5189-3p is downregulated in FLSs by BMSC exosomes and inhibits apoptosis of FLSs by downregulating BATF2 via the JAK2/STAT3 pathway. Moreover, BMSC exosome or miR-5189-3p alleviates clinical symptoms in curdlan-induced SKG mouse model of AS. Our findings may provide more insights for the molecular mechanisms of AS progression and will be helpful for the development of novel and effective treatment strategies for AS by BMSCs exosomes.

## Supplementary Material

Supplemental Material

## Data Availability

The datasets during and/or analyzed during the current study available from the corresponding author on reasonable request.
