# Supplementary material for: Bone marrow mesenchymal stem cells-derived exosomes suppress miRNA-5189-3p to increase fibroblast-like synoviocyte apoptosis via the BATF2/JAK2/STAT3 signaling pathway
Source: Bioengineered. 2022 Mar 4;13(3):6767–80. doi: 10.1080/21655979.2022.2045844 (PMC8973596; doi:10.1080/21655979.2022.2045844)
Supplement: Supplemental Material [file KBIE_A_2045844_SM8484.zip › Supplementary material S3.docx]

**Supplementary materials S1**

BATF2:

ATGCACCTCTGTGGGGGCAATGGGCTGCTGACCCAGACAGACCCCAAGGAGCAACAAAGGCAGCTGAAGAAGCAGAAGAACCGGGCAGCCGCCCAGCGAAGCCGGCAGAAGCACACAGACAAGGCAGACGCCCTGCACCAGCAGCACGAGTCTCTGGAAAAAGACAACCTCGCCCTGCGGAAGGAGATCCAGTCCCTGCAGGCCGAGCTGGCGTGGTGGAGCCGGACCCTGCACGTGCATGAGCGCCTGTGCCCCATGGATTGTGCCTCCTGCTCAGCTCCAGGGCTCCTGGGCTGCTGGGACCAGGCTGAGGGGCTCCTGGGCCCTGGCCCACAGGGACAACATGGCTGCCGGGAGCAGCTGGAGCTGTTCCAGACCCCGGGTTCCTGTTACCCAGCTCAGCCGCTCTCTCCAGGTCCACAGCCTCATGATTCTCCCAGCCTCCTCCAGTGCCCCCTGCCCTCACTGTCCCTTGGCCCCGCTGTGGTTGCTGAACCTCCTGTCCAGCTGTCCCCCAGCCCTCTCCTGTTTGCCTCGCACACTGGTTCCAGCCTGCAGGGGTCTTCCTCTAAGCTCAGTGCCCTCCAGCCCAGCCTCACGGCCCAAACTGCCCCTCCACAGCCCCTCGAGCTGGAGCATCCCACCAGAGGGAAGCTGGGGTCCTCTCCCGACAACCCTTCCTCTGCCCTGGGGCTTGCACGTCTGCAGAGCAGGGAGCACAAACCTGCTCTCTCAGCAGCCACTTGGCAAGGGCTGGTTGTGGATCCCAGCCCTCACCCTCTCCTGGCCTTTCCTCTGCTCTCCTCTGCTCAAGTCCACTTCTAA

BATF2-sh:

[SS Sequence](http://biodev.extra.cea.fr/DSIR/DSIR.php?sort=31): AGCTGAAGAAGCAGAAGAA

A[S Sequence](http://biodev.extra.cea.fr/DSIR/DSIR.php?sort=31): TTCTTCTGCTTCTTCAGCT
