# Supplementary material for: Bone marrow mesenchymal stem cells-derived exosomes suppress miRNA-5189-3p to increase fibroblast-like synoviocyte apoptosis via the BATF2/JAK2/STAT3 signaling pathway
Source: Bioengineered. 2022 Mar 4;13(3):6767–80. doi: 10.1080/21655979.2022.2045844 (PMC8973596; doi:10.1080/21655979.2022.2045844)
Supplement: Supplemental Material [file KBIE_A_2045844_SM8484.zip › Supplementary material S5.pptx]

## Slide 1
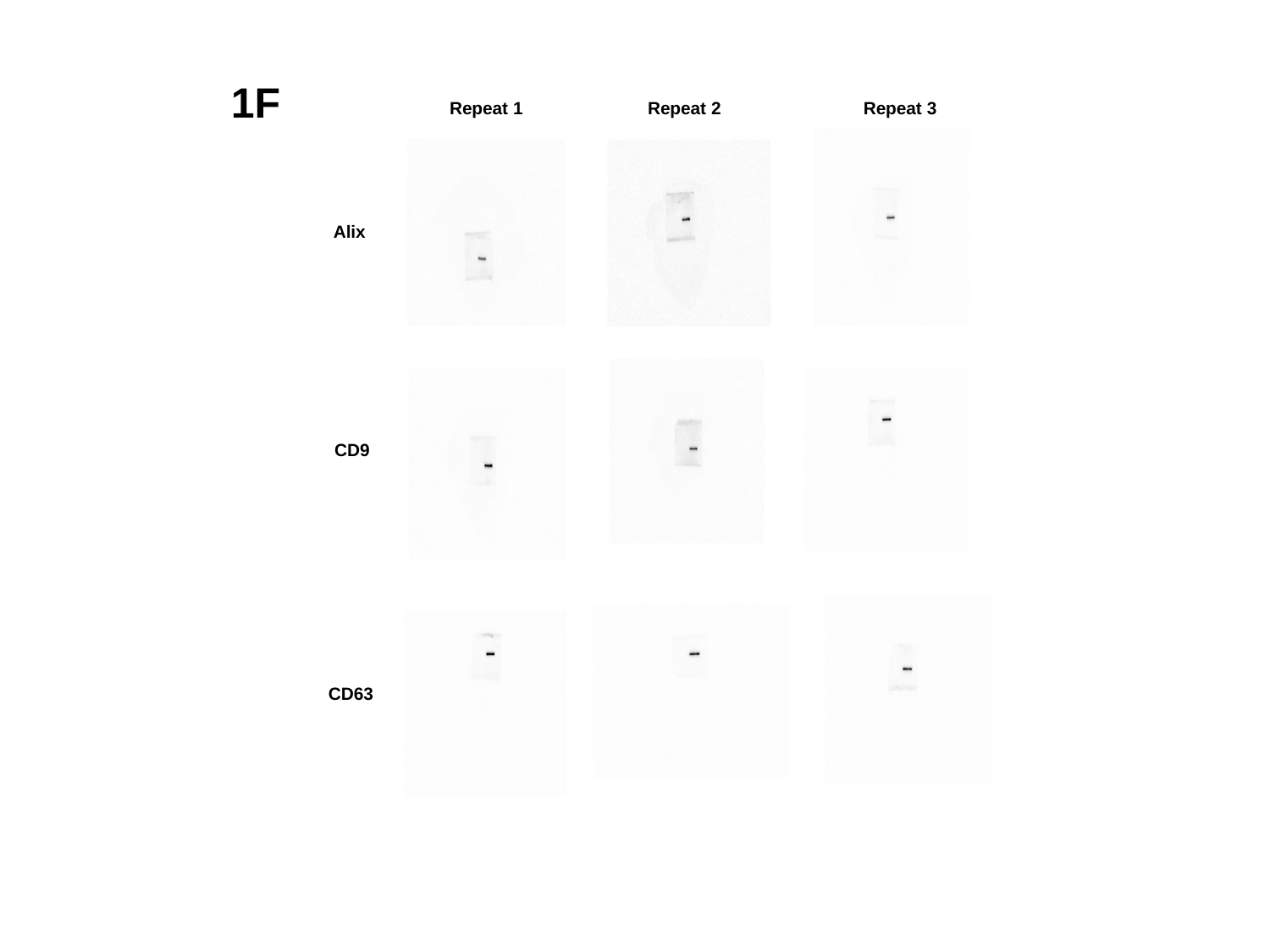

1F
Repeat 1
Repeat 2
Repeat 3
Alix
 CD9
CD63

## Slide 2
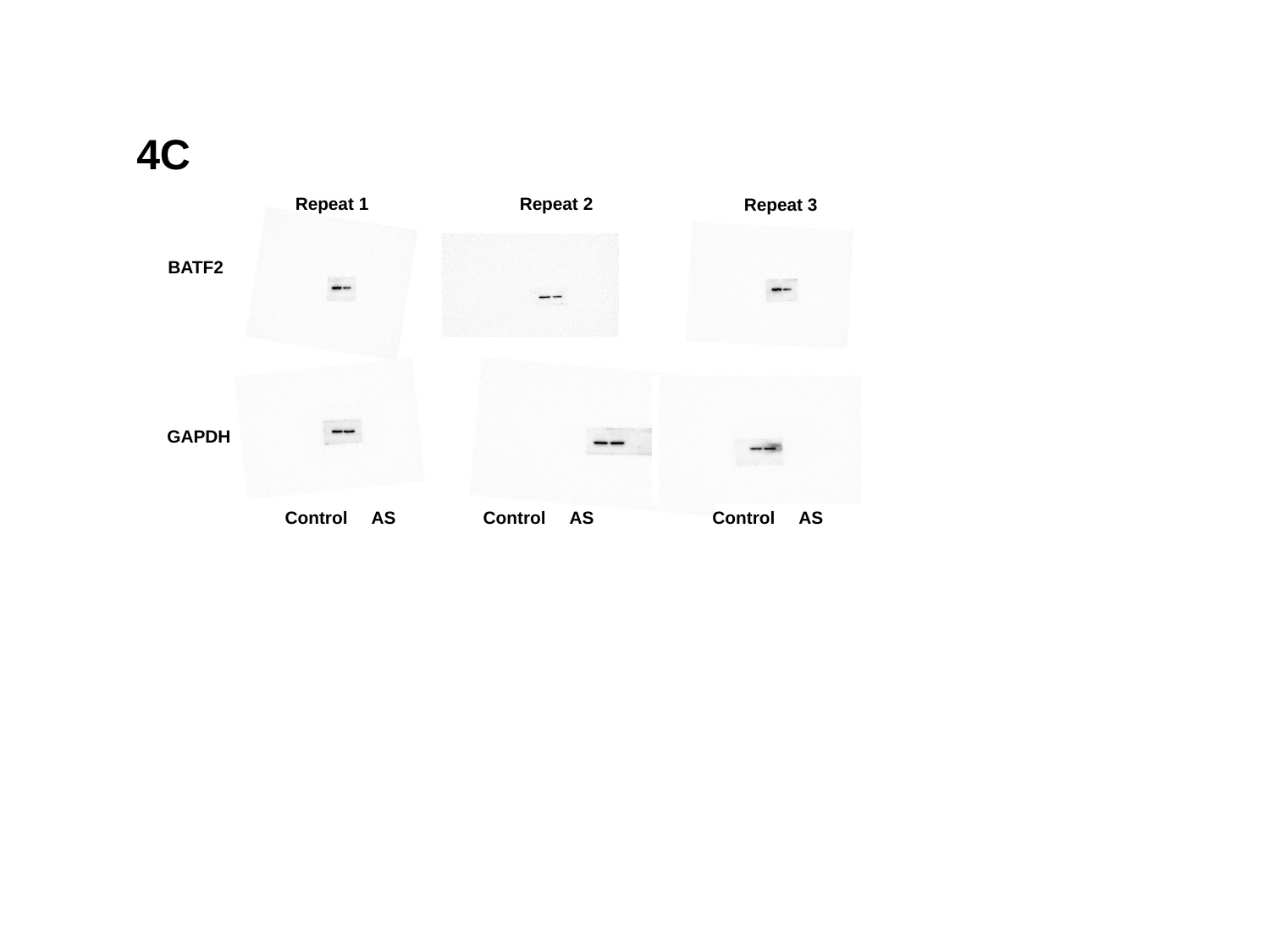

4C
Repeat 1
Repeat 2
Repeat 3
 BATF2
 GAPDH
 Control AS
 Control AS
 Control AS

## Slide 3
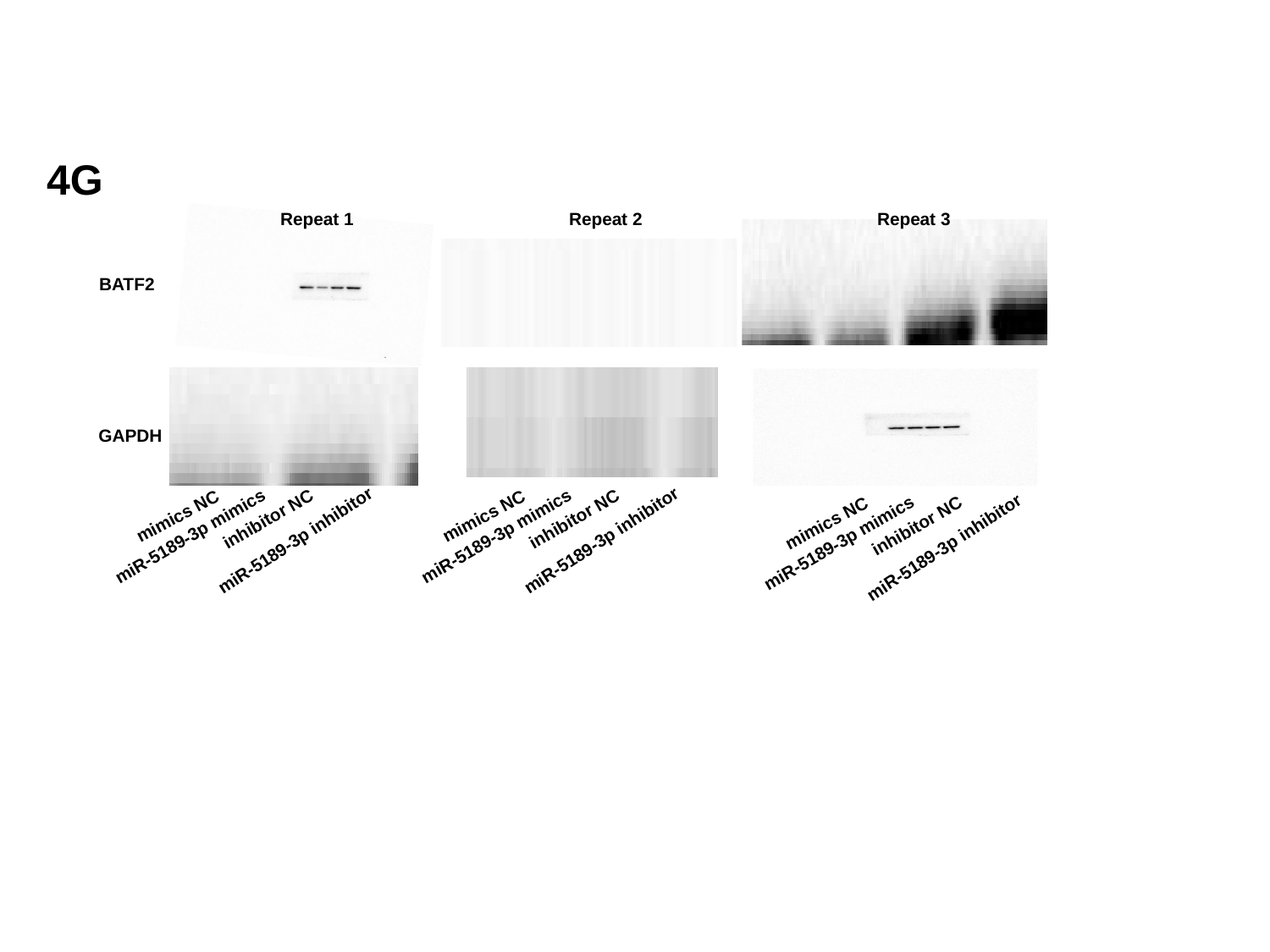

4G
Repeat 1
Repeat 3
Repeat 2
 BATF2
 GAPDH
 mimics NC
inhibitor NC
miR-5189-3p mimics
miR-5189-3p inhibitor
 mimics NC
inhibitor NC
miR-5189-3p mimics
miR-5189-3p inhibitor
 mimics NC
inhibitor NC
miR-5189-3p mimics
miR-5189-3p inhibitor

## Slide 4
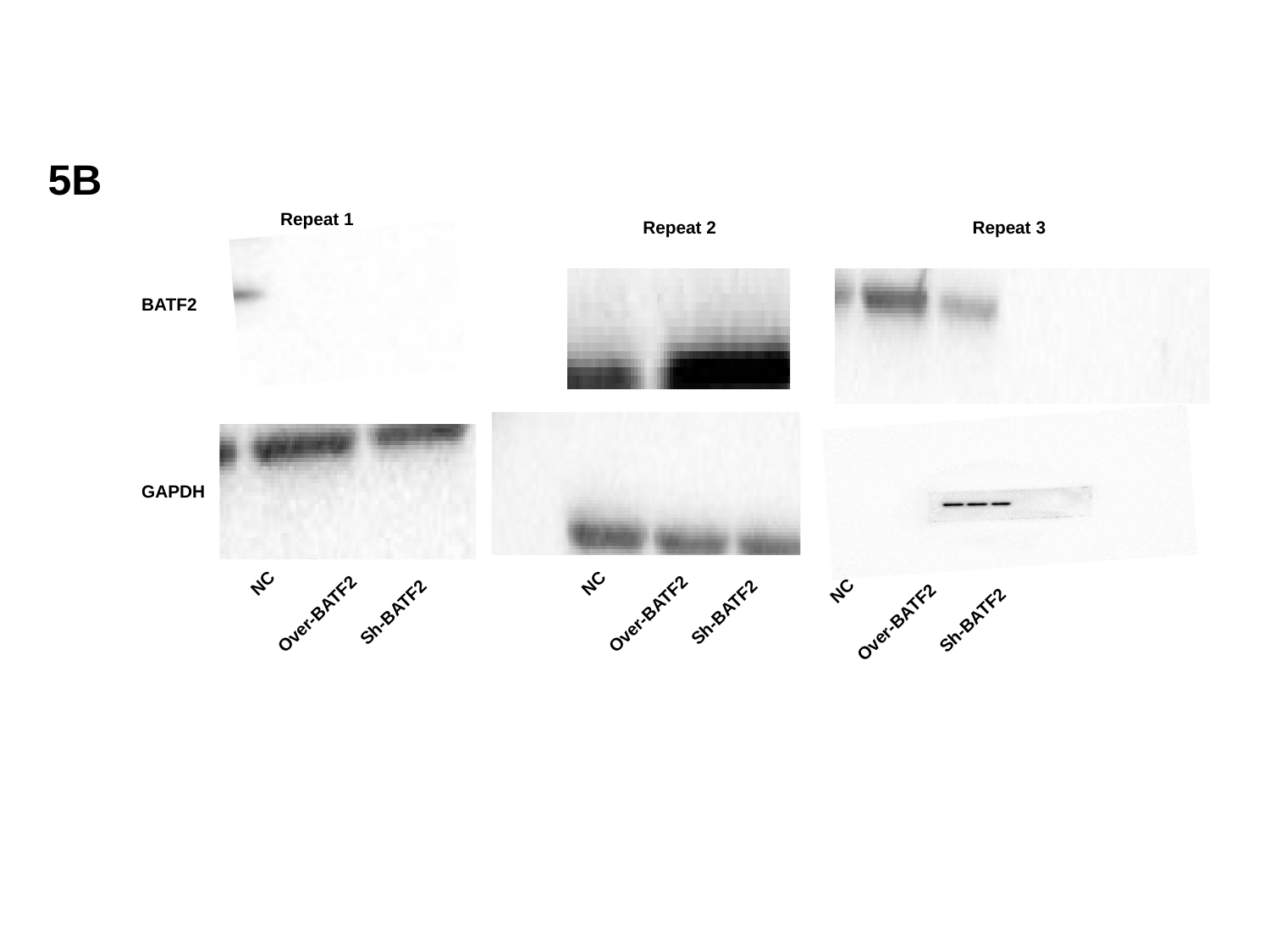

5B
Repeat 1
Repeat 2
Repeat 3
 BATF2
 GAPDH
 NC
 NC
 NC
 Sh-BATF2
 Sh-BATF2
 Over-BATF2
 Over-BATF2
 Sh-BATF2
 Over-BATF2

## Slide 5
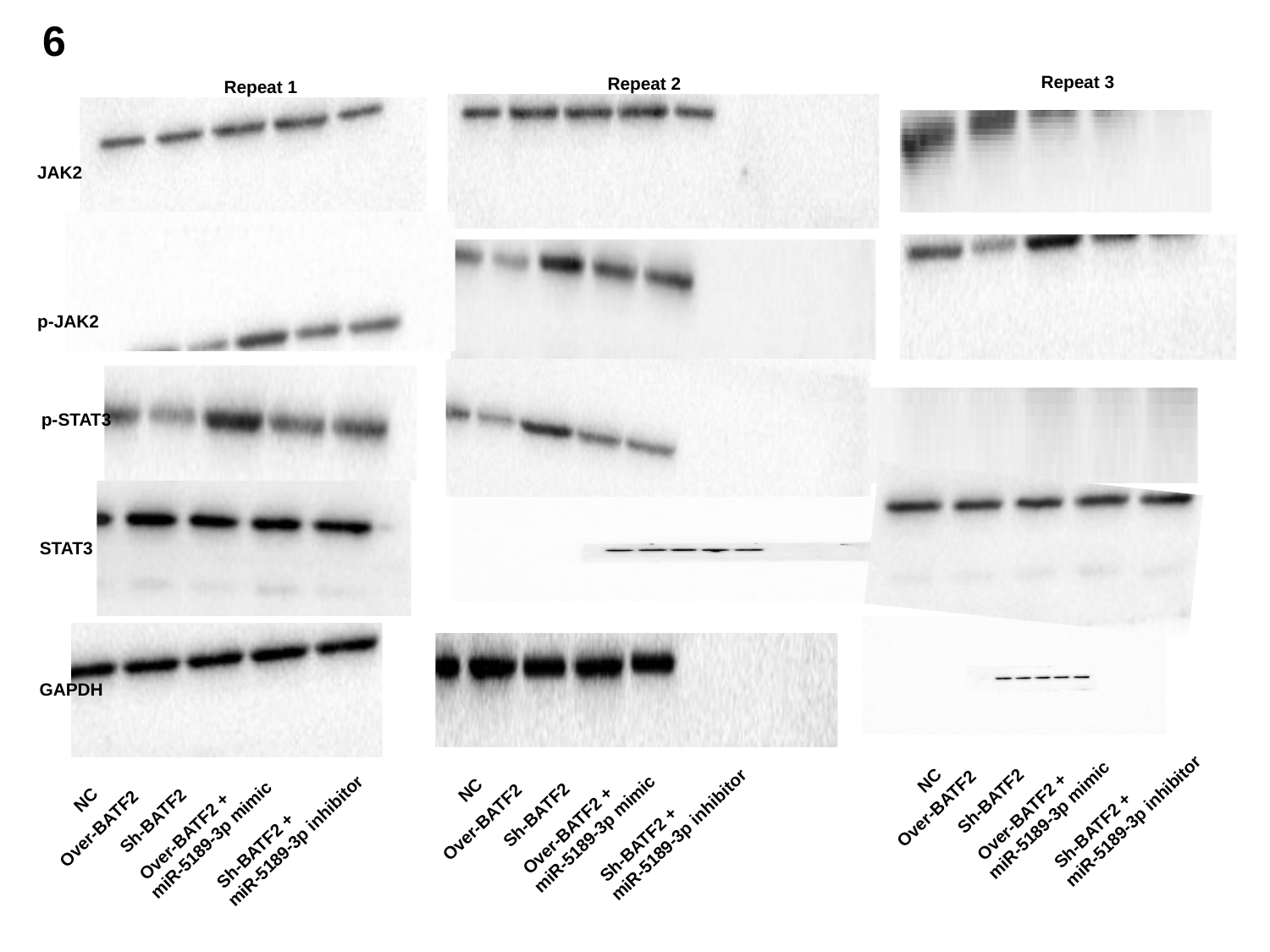

6
Repeat 3
Repeat 2
Repeat 1
JAK2
p-JAK2
p-STAT3
STAT3
GAPDH
 Over-BATF2 +
miR-5189-3p mimic
 NC
 Sh-BATF2 +
miR-5189-3p inhibitor
 Sh-BATF2
 Over-BATF2
 NC
 Over-BATF2 +
miR-5189-3p mimic
 Sh-BATF2 +
miR-5189-3p inhibitor
 Sh-BATF2
 Over-BATF2
 Over-BATF2 +
miR-5189-3p mimic
 NC
 Sh-BATF2 +
miR-5189-3p inhibitor
 Sh-BATF2
 Over-BATF2
